# War Injuries and Nurses' Well-Being: Fatigue and Sleep Quality Among Critical Care Nurses in Najran Region, Saudi Arabia

**DOI:** 10.7759/cureus.64889

**Published:** 2024-07-19

**Authors:** Samah Ramadan Elrefaey, Sameer H Hamdy, Mohammed Abdelrahman, Shaimaa Mohamed Nageeb, Randa Mohamed Abobaker, Mohmmad Alhusinat, Reem Aied Assiry, Amal H. Mohamed, Elsadig Eltaher Hamed Abdulrahman, Fatma Abdelaziz Mohammed

**Affiliations:** 1 Department of Psychiatric and Mental Health Nursing, Faculty of Nursing, Benha University, Benha, EGY; 2 Department of Community and Mental Health Nursing, Faculty of Nursing, Najran University, Najran, SAU; 3 Department of Community and Mental Health Nursing, College of Nursing, Najran University, Najran, SAU; 4 Department of Psychiatric and Mental Health Nursing, College of Nursing, University of Hail, Hail, SAU; 5 Department of Maternal and Child Health Nursing, Northern College of Nursing, Arar, SAU; 6 Department of Nursing, University of Tabuk, Tabuk, SAU; 7 Department of Nursing Administration, College of Nursing, Najran University, Najran, SAU; 8 Department of Nursing, Northern College of Nursing, Arar, SAU; 9 Department of Medical and Surgical Nursing, Faculty of Nursing, Najran University, Najran, SAU; 10 Department of Medical Surgical Nursing, College of Nursing, Najran University, Najran, SAU; 11 Department of Critical Care and Emergency Nursing, Faculty of Nursing, Cairo University, Cairo, EGY

**Keywords:** saudi arabia, war injuries, nurses in critical units, sleep quality, fatigue

## Abstract

Introduction

Critical care nurses must maintain optimal work performance. Fatigue and sleep disturbance can limit safe practice and cause negative patient outcomes. This study aimed to explore fatigue and sleep quality among critical care nurses in the war zone in Najran City, Saudi Arabia.

Methods

A cross-sectional research design was used and a convenience sample was applied to include 352 nurses working in critical units at various hospitals in Najran City, Saudi Arabia. A self-administered questionnaire containing three parts was used: demographic characteristics, the Pittsburgh Sleep Quality Index (PSQI), and the Fatigue Severity Scale (FSS).

Results

The study revealed that 232 nurses (65.9%) reported poor sleep quality. Regarding fatigue levels, 89 nurses (25.2%) reported severe fatigue and 113 (32.1%) reported moderate fatigue. Notably, caring for war-related injuries exhibited a significant positive correlation (r = 0.62, p = 0.0001). Experience correlated negatively (r = -0.47, p = 0.003) with sleep quality and fatigue scores. Most significantly, involvement in caring for war-related injuries showed a strong positive correlation (r = 0.71, p = 0.00001) with FSS scores.

Conclusions

Poor sleep quality was significantly widespread among the studied nurses. The results indicated that about one-quarter of the studied nurses reported severe fatigue, which was alarmingly prevalent among nurses. Nurses involved in caring for war-related injuries exhibited a strong positive correlation with both PSQI and FSS scores.

Recommendations

The authors recommend developing and implementing counseling and stress management programs to address the unique challenges faced by nurses caring for war-related injuries.

## Introduction

Critical care nurses encounter unique difficulties in war-torn areas that have a substantial negative impact on their physical and mental health. Their general quality of life and mental health may be affected as a result of their ongoing exposure to trauma, injuries, and stressful situations. Nursing professionals may experience psychological trauma and emotional distress as a result of directly experiencing the effects of war, such as caring for casualties and treating serious injuries [1].

In addition, the long hours and unpredictability of war environments exacerbate the symptoms of post-traumatic stress disorder. Nurses who work in hectic, high-pressure settings on a daily basis may experience sleep disturbances, nightmares, and intrusive thoughts [2].

In addition, the psychological strain of tending to patients in critical condition, observing fatalities, and navigating ethical quandaries can result in sleep disorders, moral harm, and additional psychological and physical health issues for nurses working in conflict areas. It is critical to identify and meet the mental health needs of nurses working in combat zones by giving them access to resources for mental health, enough support, and chances for self-care and debriefing. Nurses serving in war-affected areas can benefit from a supportive work environment, resilience-building techniques, and open communication, all of which can help lessen the negative effects and enhance their well-being [3].

The idea of sleep quality is complex and linked to a wide range of variables, including weariness, stress from a heavy workload, and other stressors. The quality of nursing care is seriously impacted by critical nurses' poor sleep [4]. It not only puts critical nurses' health at risk, but it also hinders their ability to perform, increases the possibility of medical errors, and reduces patient safety [5]. One of the most fundamental physiological needs, sleep, has a significant impact on both physical and mental well-being in people [6].

Fatigue is a difficult term to define and a complex idea. However, weariness is typically regarded as a multifaceted, nonspecific, multicausal, and subjective phenomenon that results from the long-term effects of social, psychological, and economic factors [7]. There are four types of fatigue: acute, chronic, mental, and physical. Workload, an increased nurse-to-patient ratio, an unsuitable working environment, and unforeseen work are factors that contribute to nurse fatigue [8,9].

Nursing is a demanding profession that requires a fair amount of mental and physical exertion. The nursing profession is full of stressful situations that can lead to low efficiency and other issues like dissatisfaction [10]. These circumstances result in psychological and physical issues that harm the nursing care system permanently [11].

This study aimed to explore fatigue and sleep quality among critical care nurses in the war zone in Saudi Arabia.

## Materials and methods

A cross-sectional research design was utilized to achieve the aim of the study. Data collection continued from June 2020 to September 2020. The study was conducted in the critical care units of three hospitals located in Najran City: King Khaled Hospital, Najran University Hospital, and Najran General Hospital. A total of 487 nurses were employed in these critical care units. To ensure that our sample was representative of the population, we employed a convenience sampling method. This method involved selecting all available nurses who met our inclusion criterion of having at least one year of experience working in critical care units. Consequently, the final sample size for the study was 352 nurses.

Data collection

The collection of data was done using a self-administered questionnaire that was translated into Arabic and comprised three sections. The first part addressed demographic profiles such as age, gender, experience, and history of caring for war injuries among the participants. The second part included the Pittsburgh Sleep Quality Index (PSQI), a 19-item self-rated questionnaire designed to assess sleep quality and disturbances over the preceding month in the study population. The questionnaire evaluates seven components: sleep duration, sleep disturbance, sleep latency, daytime dysfunction due to sleepiness, sleep efficiency, overall sleep quality, and sleep medication use. Each component is scored from 0 to 3, with a higher score indicating greater dysfunction. These component scores are summed to yield a total score ranging from 0 to 21, where higher scores indicate poorer sleep quality (0-4 indicating “good” sleep and 5-21 indicating “poor” sleep) [12].

The third part included the Fatigue Severity Scale (FSS), adapted from Valko et al. [13], which consists of nine items, each rated on a scale from one to seven (1 = strongly disagree, 7 = strongly agree). Total fatigue is calculated by summing the scores of these items, resulting in a scale range of nine to 63. Fatigue severity is categorized as follows: severe fatigue for scores greater than 42, moderate fatigue for scores between 21 and 42, and low fatigue for scores less than 21.

The researchers conducted a pilot study involving 35 nurses, which constituted 10% of the total nurses from the specified settings. The primary objectives were to assess the usability and clarity of the developed tools and to estimate the time needed for participants to complete the questionnaire. Content validity was confirmed by gathering feedback from a panel of experts in psychiatric nursing and medical-surgical nursing departments, who evaluated the format, layout, consistency, precision, and relevance of the tools.

All eligible participants were given comprehensive information about the study's goals, methods, and voluntary nature prior to enrollment. Each participant gave their informed consent, which emphasized their freedom to decline participation or to leave the study at any moment without consequence or requirement to give a reason. The study was conducted with the utmost ethical guidelines, guaranteeing the participants' privacy and confidentiality. Surveys conducted in person were used to gather the data.

Data collected from the studied sample were revised, coded, and entered using a personal computer (PC). Computerized data entry and statistical analysis were performed using SPSS version 24 (IBM Corp., Armonk, NY). Data were presented using descriptive statistics in the form of numbers, percentages, mean, and SD. Spearman and Pearson's correlation coefficients are the statistical tests used to measure the statistical relationship or association between variables.

## Results

Table 1 provides a comprehensive overview of the characteristics of the nurses studied, based on a sample size of 352 participants. The table categorizes nurses into three age groups: 20 to <30 years (112, 31.8%), 30 to <40 years (141, 40.1%), and ≥40 years (99, 28.1%). The mean age of the participants was 33.90 years, with a standard deviation of 7.95 years. The gender distribution shows that 147 nurses (41.8%) were males and 205 nurses (58.2%) were females. Regarding educational attainment, the majority of nurses hold a Bachelor of Nursing degree (252, 71.6%), followed by those with a technical high school of nursing diploma (79, 22.4%) and a smaller proportion with a postgraduate qualification (21, 6%). The distribution of years of nursing experience revealed that 135 nurses (38.3%) had less than 10 years of experience, 145 (41.2%) had between 10 and less than 20 years of experience, and 72 (20.5%) had 20 or more years of experience. The mean years of experience among the participants was 14.13 years, with a standard deviation of 4.29 years. In terms of involvement in caring for war-related injuries, 190 nurses (53.9%) reported being involved, while 162 nurses (46.1%) did not.

**Table 1:** Distribution of nurses studied according to their characteristics data (n = 352).

Items	N	%
Age (year)		
20-<30	112	31.8
30-<40	141	40.1
≥40	99	28.1
Mean ± SD = 33.90 ± 7.95		
Gender		
Male	147	41.8
Female	205	58.2
Educational level		
Technical high school of nursing	79	22.4
Bachelor of nursing	252	71.6
Master	21	6
Years of nursing experience		
<10	135	38.3
10-<20	145	41.2
≥20	72	20.5
Mean ± SD = 14.13 ± 4.29		
Involved in caring for war-related injuries		
Yes	190	53.9
No	162	46.1

Table 2 presents the distribution of the studied sample based on their quality of sleep and fatigue levels. The data include a total of 352 nurses. The table shows that out of 352 nurses, 120 (34.1%) reported having good sleep quality, while the majority, 232 (65.9%), reported poor sleep quality. Regarding fatigue levels, the table indicates that 89 nurses (25.2%) reported severe fatigue, 113 (32.1%) reported moderate fatigue, and 150 (42.7%) reported mild fatigue.

**Table 2:** Distribution of the studied sample according to the quality of sleep and fatigue level (n = 352).

Items	N	%
Quality of sleep		
Good sleep	120	34.1
Poor sleep	232	65.9
Fatigue level		
Severe	89	25.2
Moderate	113	32.1
Mild	150	42.7

Table 3 reveals the correlation analysis regarding the factors influencing sleep quality among nurses. Firstly, there exists a significant negative correlation (r = -0.55, p = 0.001) between years of experience and the PSQI score. This indicates that as nurses gain more experience, their likelihood of experiencing sleep disturbances decreases. Conversely, a weak positive correlation (r = 0.15, p = 0.08) was observed between educational level and PSQI scores. Additionally, a notable positive correlation (r = 0.45, p = 0.01) between gender and PSQI scores underscores that female nurses tend to report higher PSQI scores. Moreover, involvement in caring for war-related injuries shows a strong positive correlation (r = 0.62, p = 0.0001) with PSQI scores, indicating that nurses dealing with such injuries experience more severe sleep disturbances.

**Table 3:** Correlation between experience, educational level, gender, and caring for war-related injuries with the Pittsburgh Sleep Quality Index.

Items	R	P
Experience	-0.55	0.001
Educational level	0.15	0.08
Gender	0.45	0.01
Involved in caring for war-related injuries	0.62	0.0001

Table 4 identifies that there is a negative correlation (r = -0.47, p = 0.003) between years of experience and FSS scores, suggesting that as nurses accumulate more experience, their reported fatigue levels tend to decrease. Conversely, there is a positive correlation (r = 0.21, p = 0.06) between educational level and FSS scores. Gender exhibits a notable positive correlation (r = 0.53, p = 0.001) with FSS scores, revealing that female nurses typically report higher levels of fatigue than male counterparts, underscoring gender as a significant factor in fatigue severity among nurses. Most prominently, the strong positive correlation (r = 0.71, p = 0.00001) between involvement in caring for war-related injuries and FSS scores highlights that nurses engaged in such care experience markedly higher fatigue levels.

**Table 4:** Correlation between experience, educational level, gender, and caring for war-related injuries with the Fatigue Severity Scale.

Items	R	P
Experience	-0.47	0.003
Educational level	0.21	0.06
Gender	0.53	0.001
Involved in caring for war-related injuries	0.71	0.00001

Table 5 shows the correlation between sleep quality and fatigue among nurses, revealing a strong positive relationship. The correlation coefficient (r = 0.705) indicates a substantial association between higher levels of fatigue and poorer sleep quality. The p-value (0.000) is highly significant, being well below the threshold of 0.01. This significant correlation suggests that as fatigue levels increase, sleep quality deteriorates.

**Table 5:** Correlation between sleep quality and fatigue. ** denote a p-value < 0.01 (very significant).

Items	Sleep quality
Fatigue	r = 0.705; p = 0.000**

## Discussion

After analyzing and interpreting the collected data, the current study demonstrated that approximately two-thirds of the nurses studied had poor sleep quality, while around one-third had good sleep quality. These results are supported by the study conducted by Han et al. [14], who stated that a significant percentage of nurses experienced problems with the quality of sleep. In the same line, Chueh et al. [15] reported that two-thirds of the nurses suffered from poor sleep. Additionally, Qiu et al. [16] reported that Chinese healthcare professionals suffered from sleep troubles higher than the general population. The prevalence of poor sleep was lower in this study compared to various studies, such as the study by Segon et al. [17] in Ethiopia, which noted that three-quarters of nurses had poor sleep, and the Nigerian study by Kolo et al. [18], which reported that more than three-quarters of nurses reported poor sleep. In the healthcare system in Saudi Arabia, the availability of resources may be the rationale for the differences.

The current study revealed that there was a significant negative correlation between years of experience and PSQI score. Conversely, while a weak positive correlation was observed between educational level and PSQI score, the association was not statistically significant, because all of the highly educated nurses were newly graduated, implying that educational attainment alone may not significantly impact sleep quality. The present study was supported by the Egyptian study conducted by Omar et al. [19], who reported that older and more experienced nurses use coping mechanisms that decrease their likelihood of experiencing sleep disturbances. On the other hand, Christina and Konjengbam [20] contradicted the results of the current study.

The current study revealed that the involvement in caring for war-related injuries shows a strong positive correlation (r = 0.62, p = 0.0001) with PSQI scores, indicating that nurses dealing with such injuries experience more severe sleep disturbances. This finding is supported by Abdelrahman et al. [1], who conducted their study in Sudan. They reported that providing nursing care during the war had a negative impact on the sleep quality and mental health of nurses, consistent with our results. Similarly, Kenny and Kelley [3] also found that the ethical and emotional burdens faced by military nurses during war negatively affect their sleeping patterns and mental health, further validating our findings. These studies collectively underscore the profound impact of war-related nursing care on sleep quality, highlighting the need for targeted interventions to support the well-being of nurses in such high-stress environments.

The present study revealed significant findings related to FSS among nurses. Less than half of the nurses studied reported moderate fatigue, while one-quarter experienced severe fatigue and slightly less than one-third reported low fatigue. These results highlight the prevalence and variation in fatigue levels among nurses, reflecting the demanding nature of their work environments. The findings of this study are supported by previous research. Abdul Rahman et al. [21] conducted a study comparing psychosocial work stressors, work fatigue, and musculoskeletal disorders between emergency and critical care nurses in public hospitals in Brunei. They found that work fatigue was a prevalent issue among nurses, aligning with our observations of moderate to severe fatigue levels in a substantial portion of the nursing workforce. This comparison emphasizes the widespread nature of fatigue in high-stress nursing roles.

Additionally, Drake and Steege [22] provided further support to our findings through their interpretation of hospital nurse fatigue using latent profile analysis. Their study identified varying levels of fatigue among nurses and underscored the significant impact of work-related factors on fatigue severity. The identification of moderate to severe fatigue levels in our study is consistent with their analysis, highlighting the need for targeted interventions to address fatigue among nurses.

The analysis presented in Table 5 shows a significant correlation between sleep quality and fatigue among nurses, revealing a strong positive relationship. The correlation coefficient (r = 0.705) indicates a substantial association between higher levels of fatigue and poorer sleep quality. This finding is consistent with previous research. Çelik et al. [23] examined fatigue in intensive care nurses and found that high levels of fatigue were closely related to poor sleep quality. Their study emphasized that intensive care nurses often experience significant fatigue due to the demanding nature of their work, which in turn negatively impacts their sleep quality. Along the same line, the study agreed with the current study's results, highlighting the critical impact of fatigue on sleep health among nurses.

The current study revealed that female nurses typically report higher levels of fatigue than male counterparts, underscoring gender as a significant factor in fatigue severity among nurses. These results are supported by the study conducted by Sagherian et al. [24] and Rashidi et al. [25].

The results of this study demonstrated a significant correlation between involvement in caring for war-related injuries and fatigue severity among nurses. The strong positive correlation (r = 0.71, p = 0.00001) indicates that nurses who are engaged in caring for war-related injuries experienced markedly higher levels of fatigue. This finding aligns with previous research that has highlighted the challenges faced by healthcare professionals working in high-stress environments. Lang et al. [26] compared nurse burnout across different army hospital practice environments and found that nurses in more stressful and demanding settings, such as those dealing with war-related injuries, exhibited higher levels of burnout. This supports our finding that the intense nature of caring for war-related injuries significantly contributes to increased fatigue among nurses.

Additionally, the study by Owen and Wanzer [27] on compassion fatigue in military healthcare teams further corroborates our results. They identified that healthcare professionals exposed to the trauma and suffering of patients, particularly in military settings, are at a higher risk of experiencing compassion fatigue. This form of fatigue, characterized by emotional and physical exhaustion, directly impacts their ability to provide care and maintain their own well-being.

Limitations of the study

The cross-sectional design captures data at a single point in time, which prevents establishing causality or observing changes over time in the relationship between sleep quality and fatigue among nurses. Self-reported data collection through questionnaires may also lead to response bias, affecting the accuracy of the results. Furthermore, the study's three-month data collection period might not account for long-term trends or seasonal variations in sleep quality and fatigue among participants. The COVID-19 pandemic during the study period might have further influenced nurses' sleep quality and fatigue levels due to increased stress and workload, impacting the results.

Recommendations

To enhance the well-being and performance of nurses caring for war-related injuries, it is essential to develop tailored support systems, including mental health resources, counseling, and stress management programs. Effective workload management strategies should be implemented to mitigate burnout and reduce fatigue levels, ensuring adequate staffing, providing opportunities for rest, and optimizing work schedules. Resilience-building and coping mechanism training should be offered to help nurses manage stress effectively and improve overall well-being. Special attention should be given to female nurses, recognizing their higher fatigue levels and poorer sleep quality, through gender-specific interventions aimed at supporting their health and well-being. The experience of senior nurses should be utilized by involving them in mentoring roles to support less experienced staff in coping with stress and improving resilience. Finally, regular assessments of sleep quality and fatigue levels among nurses should be conducted to promptly identify issues and implement necessary interventions.

## Conclusions

The study conducted a thorough examination of sleep quality and fatigue levels among nurses. The results revealed about one-third reported having good sleep quality, while the majority reported poor sleep quality. Regarding fatigue levels, the results indicated that about one-quarter of the studied nurses reported severe fatigue, one-third reported moderate fatigue, and the rest reported mild fatigue. Key findings included a significant negative correlation between years of experience and both the PSQI and FSS scores. Conversely, a weak positive correlation was observed between educational level and both PSQI and FSS scores. Female nurses reported higher PSQI and FSS scores compared to males, indicating poorer sleep quality and higher fatigue levels, respectively. Nurses involved in caring for war-related injuries exhibited a strong positive correlation with both PSQI and FSS scores.
